# Validation of a Simulation Methodology for Thermoplastic and Thermosetting Composite Materials Considering the Effect of Forming Process on the Structural Performance

**DOI:** 10.3390/polym12122801

**Published:** 2020-11-26

**Authors:** Lorenzo Sisca, Patrizio Tiziano Locatelli Quacchia, Alessandro Messana, Andrea Giancarlo Airale, Alessandro Ferraris, Massimiliana Carello, Marco Monti, Marta Palenzona, Andrea Romeo, Christian Liebold, Salvatore Scalera, Alberto Festa, Paolo Codrino

**Affiliations:** 1 Mechanical and Aerospace Engineering Department Italy, Politecnico di Torino, 10129 Turin, Italy; lorenzo.sisca@polito.it (L.S.); alessandro.messana@polito.it (A.M.); andrea.airale@polito.it (A.G.A.); alessandro.ferraris@polito.it (A.F.); massimiliana.carello@polito.it (M.C.); 2 Proplast, 15122 Alessandria, Italy; marco.monti@proplast.it (M.M.); marta.palenzona@proplast.it (M.P.); andrea.romeo@proplast.it (A.R.); 3 DYNAmore GmbH, 70565 Stuttgart, Germany; christian.liebold@dynamore.de; 4 DYNAmore Italia S.r.l., 10122 Turin, Italy; salvatore.scalera@dynamore.it; 5 SFC Compositi S.r.l., 10098 Rivoli, Italy; alberto.festa@sfccompositi.it (A.F.); paolo.codrino@sfccompositi.it (P.C.)

**Keywords:** LS-DYNA, envyo, process simulation, thermoforming, thermostamping, thermoplastic composite, thermosetting composite, mapping, structural simulation, composite forming

## Abstract

This research work investigated the influence of the press molding manufacturing process on the mechanical properties, both for thermoplastic and thermosetting fiber reinforced composite materials. The particular geometry of the case study, called Double Dome, was considered in order to verify the behavior of the Thermoplastic and Thermosetting prepreg in terms of shell thickness variation and fibers shear angle evolution during the thermoforming process. The thermoforming simulation was performed using LS-DYNA^®^ Finite Element Analysis (FEA) code, and the results were transferred by Envyo^®^, a dedicated mapping tool, into a LS-DYNA^®^ virtual model for the structural simulation. A series of Double Dome specimens was produced with industrial equipment, and a bending experimental test was been carried on. Finally, a numerical-experimental correlation was performed, highlighting a significant forecast of the mechanical properties for the considered component.

## 1. Introduction

In recent years, the world is going through a period of radical change driven by greater attention to environmental impact of human activities. Especially in the automotive field, new mobility paradigms and innovative vehicle construction and powertrain are needed to cope with this global issue. Lightweight vehicles focalize a particular attention because of the direct effect on the fuel consumption: Sinha and Tyagi (2019) indicate that a 50% increase in vehicle mass results in an increase of approximately 60% in fuel consumption [1], and Mayyas et al. (2012) assert that a 10% reduction in vehicle weight translates to a 5% increase in miles per gallon [2]. A promising strategy to reduce the vehicle mass is the use of innovative lightweight materials, like polymer composite materials. For example, Brooks et al. (2000) report that Volkswagen realized a front-end panel for Golf with Glass Mat Thermoplastic (GMT) [3]. Al-Qureshi (2001) tested a leaf spring made in glass fiber reinforced plastic [4].

Inside the composite material, the ceramic fiber is the reinforcement and gives the high specific strength and stiffness, the polymeric matrix gives the low density and the capability of the material to be formed in a large variety of industrial processes. In particular, Yao et al. (2018) assert that the thermoplastic polymers are characterized by the lack of a curing stage, with less hazardous chemical compositions and more recycling possibilities and mass production processes, in comparison with conventional thermosetting polymers [5]. On this topic, Messana et al. (2017) highlighted the greater resistance of thermoplastic composites to hygrothermal aging instead of thermosetting ones, studying both the thermo-mechanical properties and the chemical composition degradation [6]. The automotive industry has already a great number of applications with thermoplastic composites; for example, Friedrich and Almajid (2013) report that the glass fiber in PA6 matrix is used in a transverse support beam for Porsche and in a bumper structure for BMW [7].

In general, automotive requires highly automated and robust production technologies, this makes necessary to investigate the consequences of the production process in order to calibrate and stabilize it correctly, as stated by Virgillito et al. (2019) [8]. For these reasons, Henning et al. (2019) assert that the development and the knowledge of forming technologies, like thermoforming, and their influence on the mechanical properties of the final product are of great interest [9]. The potential of the press molding technique is high both for organosheets and woven thermosetting composites [10], but it has some important differences due to the properties of the polymeric matrix. The thermoforming process for organosheets can be described in the following five stages:

- the laminates are cut in a 2D shape, which normally is the result of a trial and error procedure,
- preform is then heated generally by an infrared oven until proper temperature to ensure the formability of the polymer matrix,
- a robotic arm moves the hot laminate into the press mold,
- the mold closes and a pressure is applied to consolidate the composite part while the laminate temperature decreases, and
- finally, the part is demolded and kept to stability in air, and then it is analyzed with non-destructive techniques for quality control check.

The press molding process for the woven thermosetting composite laminates is very similar to the thermoplastic one, but it requires the following specifications:

- the thermosetting resin has to be chemically designed for press molding, with a fast curing phase of some minutes, instead of some hours as it happens for the traditional autoclave process,
- the prepreg has to be stored at −20 °C and heated in the mold to facilitate the handling and avoid the degradation,
- the low viscosity of the thermosetting resin at the forming temperature leads to the use of vertical press machines with lower load capacity, instead of the horizontal press machines used for the thermoplastic manufacturing, and
- the mold has not to be cooled down within the process cycles because of the thermosetting composite consolidation inside the mold after the correct curing time.

The virtual simulation of the composite during this process can offer several advantages for the forecasting of the manufact behavior, especially for the localization of the critical zones. In particular, Wang et al. (2013) simulated the thermoforming of satin/ polyetheretherketone (PEEK) prepregs and they studied the distribution of the shear angle of the fibers on a simple shape [11]. Guzman-Maldonado et al. (2016) simulated the thermoforming of a thermoplastic prepreg with a visco-hyperelastic model, and they obtained an experimental-numerical correlation with the shear angle of the fibers [12]. Thermoforming process is also significantly affected by temperature. Dörr et al. (2019) developed a coupled thermomechanical approach for thermoforming simulations, which allows prediction of some defects impossible to evaluate without this model [13]. Moreover, Kuhtz et al. (2018) characterized the behavior of thermoplastic composites at the forming temperature and determined that thermoforming process is influenced by shear, tensile, bending, and transverse compression properties of the material at this temperature [14]. In particular, the principal deformation mechanisms that influence thermoforming process are intra-ply shearing, shear-tension coupling, bending, and fiber tension [15]. For this reason, it is important to evaluate the mechanical properties of the material, in particular, the shear properties of fabric, that were characterized by Lee et al. (2008) [16], and the tool-ply friction behavior, that was studied by Fetfatsidis et al. (2013) [17].

The aim of this paper was to investigate on glass and carbon organosheets and carbon epoxy composites the influence of the thermoforming process on the final mechanical properties for the case study of Double Dome. In a previous work, Carello, Amirth et al. (2017) considered a simple case study called Procomp, but it did not allow an in-depth evaluation of the fiber shear deformation [18,19]. The Double Dome has a more complex shape, and it was already investigated by Ford Research Lab (2004), in which it simulated a Twintex (glass fiber reinforced polypropylene) Double Dome thermoforming, and it evaluated the shear angle of the Double Dome after the simulation [20]. The same analysis was conducted by Lee et al. (2010) using a glass fiber plain reinforced polypropylene [21] and by Khan et al. (2010) [22]. The curvatures of the Double Dome are suitable to verify the shear behavior of thermoplastic composites during the thermoforming process and all its consequences on the final surface quality, as stated by Xiong et al. (2019) [23]. Komeili and Milani (2016) also used the Double Dome shape in order to study the effect of shear tension coupling in forming simulation of woven fabric reinforcements [24].

**Figure 1:**
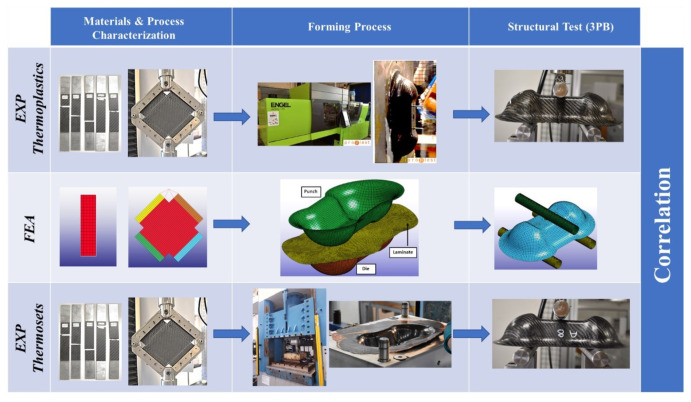
Activity flowchart for the numerical-experimental correlation of Double Dome.

The flowchart of the activity is reported in Figure 1. The selected materials were characterized, both at room temperature and both in the forming conditions, using advanced experimental setup. Specific Finite Element Analysis (FEA) models were realized on the FEM software LS-DYNA for the virtual correlation of the specimens, and the results were applied in a thermoforming simulation. A three-point bending test (3PB) was used to understand the effective structural resistance of the Double Dome. To consider the thermoforming effects, the deformed mesh parameters were transferred to the structural model using a proper mapping tool.

## 2. Materials and Methods

### 2.1. Materials Specifications

In order to carry on this study, the following industrial organosheets and prepregs have been chosen:

- TEPEX^®^ Dynalite 104-RG600(x)/47% by Bond-Laminates (Brilon, Germany), a Polypropylene organosheet reinforced with E-Glass 600 g/m^2^ Twill 2/2 balanced woven, here called “GF/PP”;
- TEPEX^®^ Dynalite 201-C200(x)/45% by Bond-Laminates (Brilon, Germany), a Polyamide 66 organosheet reinforced with Carbon 200 g/m^2^ Twill 2/2 balanced woven, here called “CF/PA66”;
- GG240T-FF-IMP509 by Angeloni, (Venice, Italy) an Epoxy prepreg with Carbon 240 g/m^2^ Twill 2/2 balanced woven, here called “CF/EP—Angeloni”;
- LTM26 by Solvay (Bruxelles, Belgium), an Epoxy prepreg with Carbon 285 g/m^2^ Twill 2/2 balanced woven, here called “CF/EP—Solvay”.

**Figure 2:**
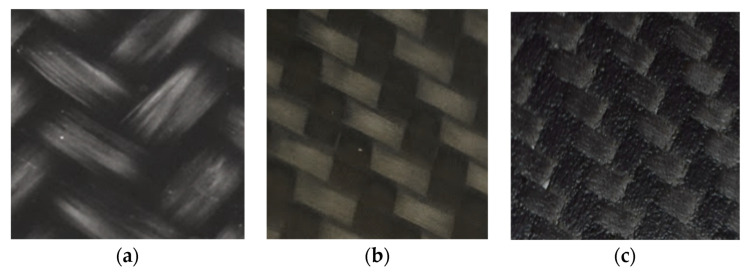
Surface detail of the chosen laminates (25 × 25 mm^2^): GF/PP (**a**), CF/PA66 (**b**), CF/EP (**c**).

The reason to use these materials is the industrial availability of Bond-Laminates organosheets as thermoplastic composite laminates, ready to be thermoformed in a wide range of thickness (number of layers). For the thermosetting prepregs, the choice was made on the Angeloni and Solvay Epoxy solutions for the press molding process, selecting the same carbon fabric of Bond laminates for a best comparison. The Twill 2/2 woven was chosen, both for glass and carbon fabrics, because of the balanced behavior. Figure 2 reports a surface detail of the three kind of the composite laminates. The black color of the materials is due to the carbon black filler in the PP matrix for GF/PP, and to the CF fiber for CF/PA66 and CF/EP.

Each composite material requires a different treatment in order to reach the best performance after the manufacturing process, mostly dependent on the polymeric matrix curing/transformation. In particular, thermosetting composites have a crosslinking reaction, on the contrary organosheets undergo a physical consolidation after cooling. Table 1 reports the values of Temperature, Pressure, Cycle Time adopted for the consolidation of the chosen materials. It has to be noted that the Cycle Time value of the CF/EP for press molding process is drastically lower than the one of traditional autoclave vacuum-bag, 5/8 min compared to 4 h. This is due to the different curing process provided from the Epoxy prepreg producer in terms of chemical matrix development. A horizontal press is used for the thermoplastic composites because it is necessary to reach high pressures that cannot be reached by the vertical press.

### 2.2. Static Tests Characterization

**Figure 3:**
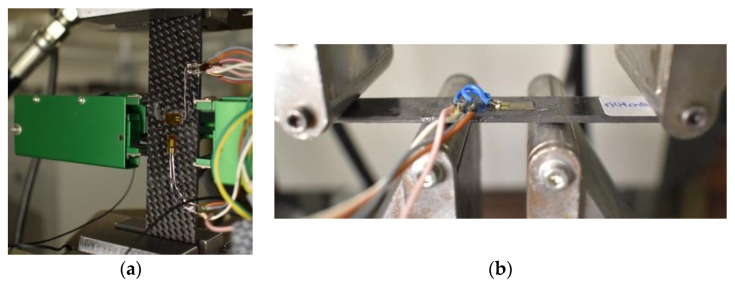
Experimental setup of Tensile test (**a**) and Bending test (**b**).

The static characterization was conducted at the Mechanical and Aerospace Engineering Department (DIMEAS) of Politecnico di Torino on the servo-hydraulic dynamometer (Instron 8801) (Norwood, MA, USA) with a 100 kN load cell. The samples were tested with the specifications reported in Table 2, according to ASTM (American Society for Testing and Materials International) standards for the composite materials. For the bending test, four cylinders of diameter 12 mm have been set at distance of 27 and 81 mm, respectively, for the bottom and top side. Figure 3 reports the experimental setup for Tensile test and Bending test, with the detail of the Epsilon 8560 clip-gage extensometer for the averaged value of axial and transversal strain and the HBM 350 Ohm strain gauges for the measure validation.

### 2.3. Process Tests Characterization

A process characterization is necessary to understand the behavior of the laminate during the production process. Therefore, a series of experimental tests have been setup at the forming temperature to investigate the intra-ply and tool-ply behavior and with the objective to set properly the process simulation.

#### 2.3.1. Intra-Ply Evaluation (Tensile and Shear Tests)

The intra-ply mechanical behavior of the composite material is greatly influenced from the properties of the fiber reinforcement. Therefore, a glass fabric 600 g/m^2^ and a carbon fabric 240 g/m^2^ were studied at room temperature without matrix resin contribution. A Tensile test, according to ASTM D5035, and a Shear test, with a proper Trellis frame, were carried on using a Zwick/Roell Z010 dynamometer (Ulm, Germany), located at Proplast and equipped with a 10 kN load cell.

**Figure 4:**
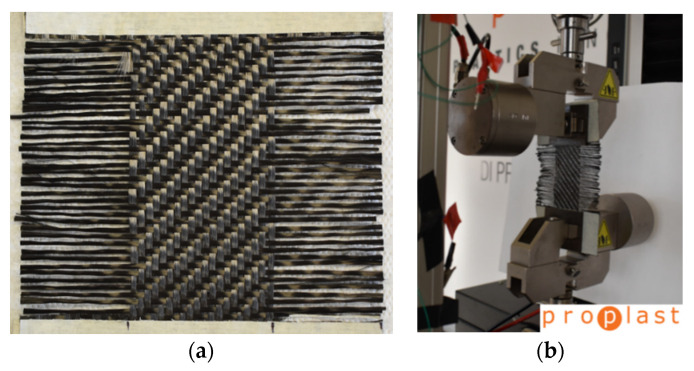
Detail of CF Specimen (**a**) and experimental setup (**b**) for Tensile test on Dry fabric for Intra-ply evaluation.

For the Tensile test the fabric specimen has been prepared from a 200 × 10 mm^2^ fabric piece. The central zone of 85 × 45 mm^2^ was obtained pulling out the external fiber strands. Tw o grip zones were covered by a paper tape in order to handle better the specimen and to spread the grip force during the test. The pictures of a specimen and the experimental setup are reported in Figure 4. The testing machine moves in Tension mode at a constant speed of 2 mm/min until the fiber breaks.

**Figure 5:**
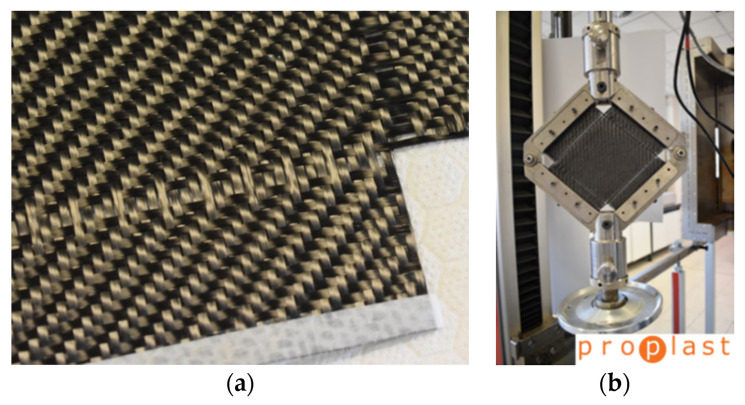
Detail of CF Specimen (**a**) and experimental setup (**b**) for Shear test on Dry fabric for Intra-ply evaluation.

The pure shear behavior between fibers of the same ply inside the mold is obtained in the plane with an especially designed Trellis frame. For the Shear test, the fabric specimen has been prepared from a 200 × 200 mm^2^ fabric piece. The central zone of 110 × 110 mm^2^ was obtained pulling out two fiber strands at the edges of the four grip zones. The pictures of a specimen and the experimental setup are reported in Figure 5. The testing machine moves in Tension mode at a constant speed of 10 mm/min from 90 to 25° angle of the Trellis frame.

#### 2.3.2. Tool-Ply Evaluation (Friction Test)

**Figure 6:**
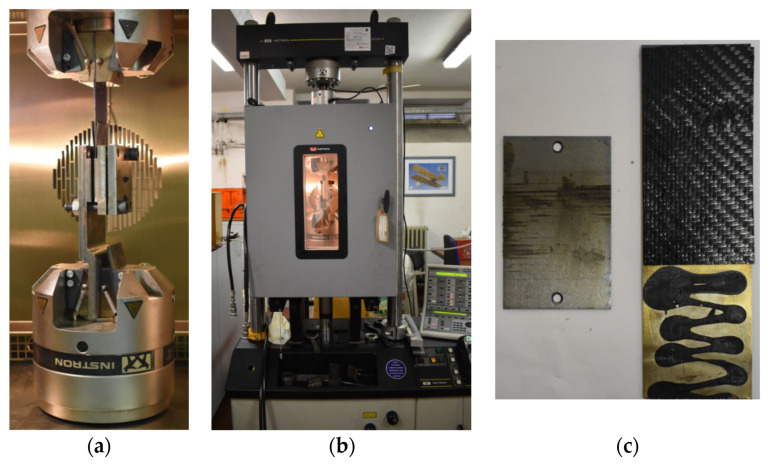
Tool-ply test: experimental setup (**a**), climatic chamber (**b**), specimen (**c**).

The tool-ply test was performed with a particular device appositely designed for this activity, in order to evaluate the static and dynamic friction coefficients necessary for the thermoforming simulation. Four shoulder bolts at the edges of the plate allow the homogeneous pressure distribution in the normal axis. The test was conducted using an Instron 8516 dynamometer, with a 100 kN load cell, equipped with air-conditioned oven that works in the range of −40 °C to 350 °C. In Figure 6, the test configuration is reported, with the picture of a specimen.

The specimen consists in two parts: two composite plates glued on the two sides of a steel plate and a steel support in contact with composite. The relative movement between composite and steel support at the forming temperature, necessary for the frictional coefficient evaluation, is provided by the Tension mode of the dynamometer crosshead. The test speed was set at 100 mm/min. The sensors used are a PT100 thermo-resistance (Milano, Italy) for the temperature and a TEKSCAN FlexiForce HT201 piezoelectric sensor (South Boston, MA, USA) for the clamping pressure. All the sensors weren controlled using a National Instruments cDAQ device (Austin, TX, USA).

The frictional coefficient µ was calculated by the Equation (1), where: F is the longitudinal force measured by the load cell of the dynamometer, and N is the clamping force normal to the specimen measured by the piezoelectric load cell.

(1) $$\mu =\;\frac{F}{N}$$

### 2.4. Thermoforming of Double Dome

#### 2.4.1. Horizontal Press

**Figure 7:**
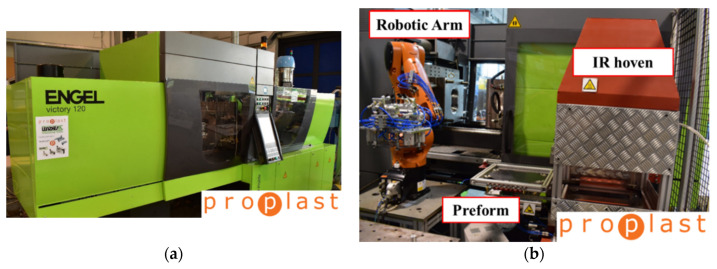
Engel Victory 500/120 press (**a**) and thermoforming equipment (**b**).

The thermoforming process for organosheet TEPEX^®^ (GF/PP and CF/PA66) was conducted on the horizontal injection press Engel Victory 500/120 (Schwertberg, Austria) reported in Figure 7.

**Figure 8:**
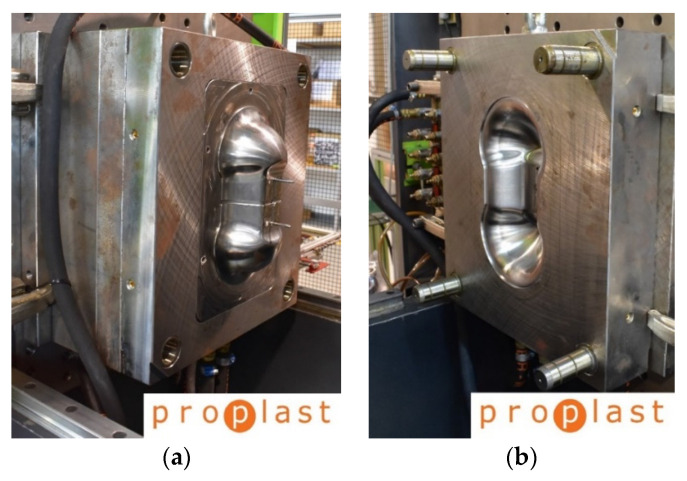
The mold punch (**a**) and the mold die (**b**).

In the pre-heating step, the laminate is taken by a Kuka anthropomorphic robot (Augusta, Germany) and placed under IR oven, where the preform is left for enough time to reach the forming temperature indicated in Table 1. The preform temperature is monitored by IR pyrometer during the oven stage and before the molding closure. The robot moves the heated laminate into the mold. A negligible cooling was noted during this operation. The correct position in the mold is ensured by two support pins, that fit into two holes previously cut in the laminate. The mold is in aluminum, and it constituted two parts: the mold core called “punch”, that is the moving part, and the mold cavity called “die”, that is the static part. The mold is heated at 40 °C to allow the laminate to be formed and cooled. The mold is opened, and the manufact is extracted. No more operations are needed. Figure 8 shows the mold punch and the mold die.

#### 2.4.2. Vertical Press

**Figure 9:**
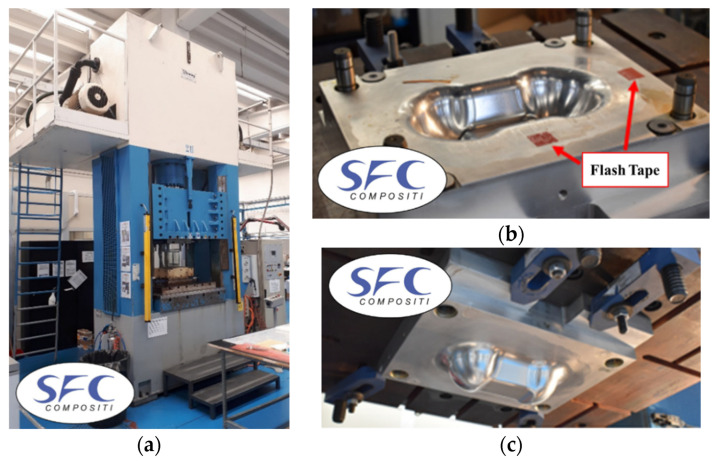
Vertical press Sick 30-FGS (**a**), Double Dome mold: top (**b**) and bottom (**c**).

A vertical press Coming Sick 30-FGS (Piacenza, Italy) was used to thermoforming thermosetting composites (Angeloni and Solvay, for this work). Figure 9 reports the vertical press located in SFC Compositi S.r.l. (Rivoli-Torino) and the aluminum Double Dome mold.

**Figure 10:**
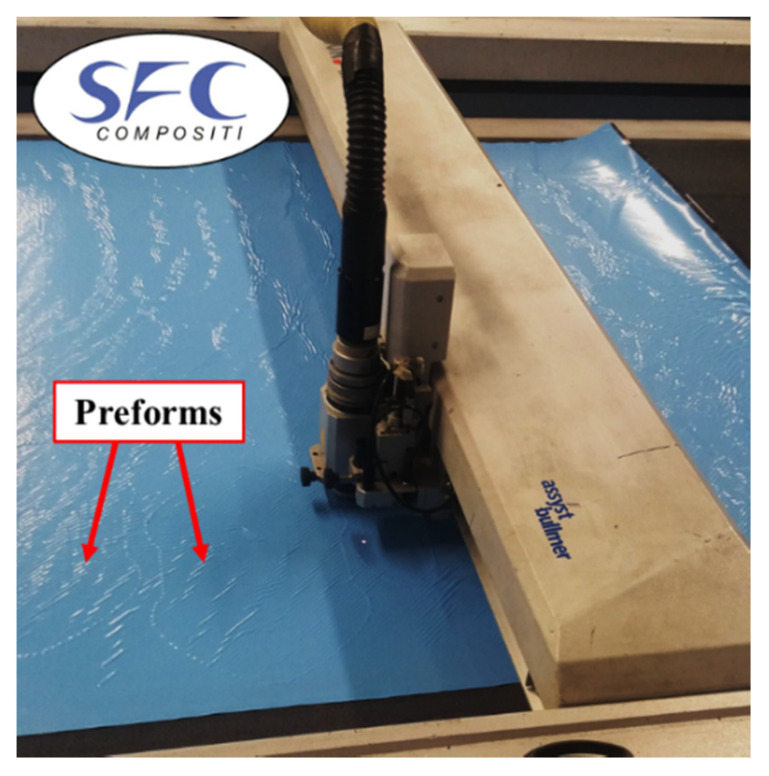
Cutting of Double Dome preforms.

The prepreg has been cut with an automatic cutter Zund M-1600 CV (Altstätten, Switzerland) reported in Figure 10. The preform is manually positioned at the room temperature (23 °C) on the lower part of the mold, with the reference of two flash tapes for alignment. The mold heating is provided by two electric heated plates and controlled in order to reach a constant temperature of 140 °C, which is suitable for the laminate thermoforming. The vertical press closes the mold until the stoppers applying the cycle pressure. At the end of the forming process, the milling is needed to remove resin overflow and to obtain the final geometry.

### 2.5. Bending Test on Double Dome

**Figure 11:**
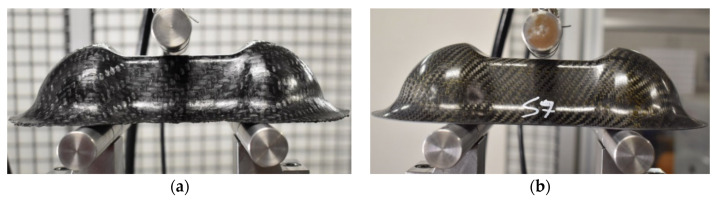
Double Dome specimens in GF/PP (**a**) and CF/EP-Solvay (**b**) before the bending test.

Three Double Dome specimens for each material, produced by thermoforming process on plates of 1 mm thickness (4 layers), were subjected to a three-point bending test in order to evaluate the mechanical properties of the part. The three steel support bars were designed for Double Dome testing, with a length of 200 mm, a diameter of 25 mm, and a lower span of 120 mm. The tests were conducted on the Instron 8801 servo-hydraulic dynamometer with a 100 kN load cell at Politecnico di Torino (DIMEAS). The sample has been tested with the speed of 2 mm/min, and the average trend of the Force-Displacement was calculated. Figure 11 reports the pictures of Double Dome specimens before the bending tests.

### 2.6. Quality Check on Double Dome

**Figure 12:**
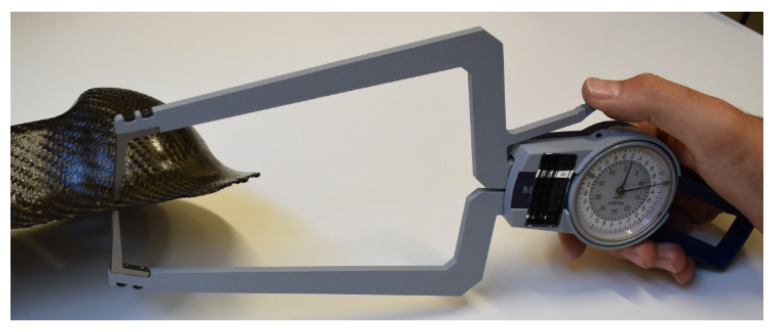
Experimental measurement of wall thickness for GF/PP Double Dome.

The wall thickness on the Double Dome samples was experimentally measured using a Mitutoyo long-arms mechanical thickness gauge (Figure 12), with a maximum range of 0–50 mm and a precision of 0.05 mm. The measurements were compared with the thermoforming simulation results.

## 3. Virtual Simulation on LS-DYNA

### 3.1. Static Tests Simulation

**Figure 13:**
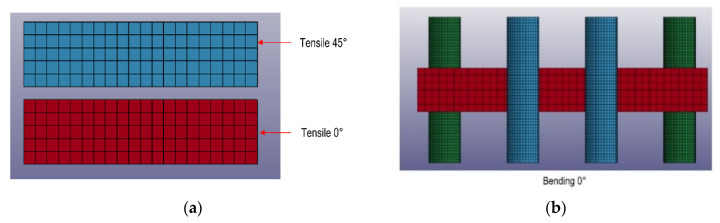
Finite Element Analysis (FEA) models of the specimens for material card setting: (**a**): Tensile specimens, (**b**): Bending specimen.

A proper material card setting is fundamental to obtain the best correlation between the experimental behavior and the FEA virtual simulations. The setting procedure was conducted reproducing the specimens of static characterization on LS-DYNA (Figure 13). The keyword *MAT_LAMINATED_COMPOSITE_FABRIC (MAT_058) was used because specific for composite laminates. In the FEA models, only the gauge areas of the specimens were reproduced, using shell meshes of 5 mm element size. For the bending test, the steel bars were meshed with 1-mm element size and modeled with *MAT_RIGID (or MAT_020). The cylinder span has been set at 81 mm for bottom cylinders and at 27 mm for the top cylinders.

### 3.2. Process Tests Simulation

**Figure 14:**
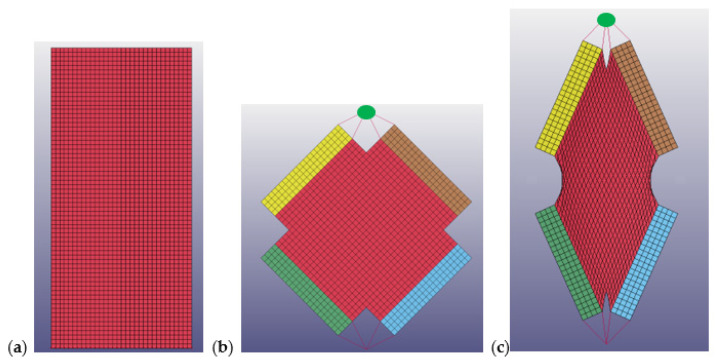
Virtual models of Tensile test (**a**) and Shear test: start point (**b**) and end point (**c**).

Intra-ply tests (Tensile and Shear) were reproduced on LS-DYNA in order to correlate the mechanical properties of the dry fabric with the behavior of the laminate during the process simulation. The material model selected for this simulation is **MAT_REINFORCED_THERMOPLASTIC (or MAT_0249), specially prepared for thermoforming models. The zero value has been imposed for resin data in the card (Young Modulus, Poisson’s ratio) in order to reproduce only the dry fabric for these tests. The size of the mesh is 2 mm for the Tensile test and 4 mm for the Shear test to obtain the best balance between simulation reliability and calculation time. In Figure 14, the virtual models for intra-ply tests are reported. For the Tensile test (a), only the gauge length of the specimen without the transverse fibers that do not work during the experimental test were reproduced. For the Shear test (b), the grips were reproduced and connected with the application load point (green point) with rigid bodies to simulate the same connections of the experimental test. The load was applied only on the green point, and the shear deformation involves only the fabric (red part), in accordance with the experimental test (c). The experimental stress-strain curves of tensile and shear test was included directly in MAT_0249 in order to reproduce the experimental results and set-up the card material for thermoforming simulation.

### 3.3. Thermoforming Simulation

**Figure 15:**
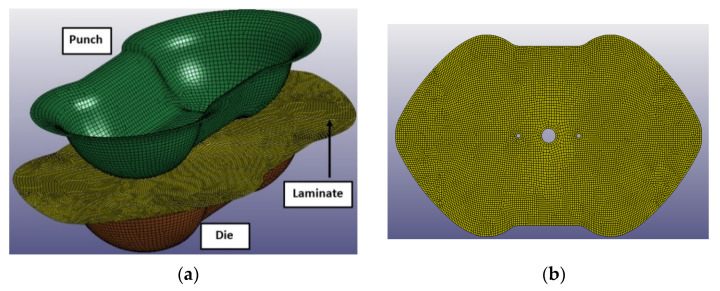
Double Dome thermoforming FEA model (**a**), Double Dome laminate mesh (**b**).

The thermoforming process was reproduced with a FEA model on LS-DYNA, in which the Double Dome is completely molded passing from a 2D to a 3D shape. In particular, the virtual model is composed by three parts: the Double Dome 2D preform, the mold core, and the mold cavity. Figure 15 reports the virtual model of thermoforming process and the laminate meshed in all details mainly with quadrilateral shell elements of size 2 mm. The laminate shape was optimized with the aid of the thermoforming process simulation, in order to avoid the classical material waste typical of the trial and error procedure. The mold closing time was set on the simulation, in order to obtain the same mold closing velocity of the real experiment. No mass scaling was implemented.

The card material used is *MAT_REINFORCED_THERMOPLASTIC (or MAT_0249), properly developed for forming simulations, based on the dry fabric tension and on the composite laminate shear. The assumed temperature of the thermoforming simulation is the process temperature for each material indicated in Table 1. The parameters of card material MAT_0249 were set using the values obtained at this temperature from the datasheet for polymeric resins (Young modulus and Poisson’s ratio) and from the process tests for dry fabrics. Intra-ply shear locking phenomena is governed by the card material with the parameters obtained from Trellis intra-ply shear test. The adopted technique is full integration. In order to replicate the reality, the mold core called punch is the moving part and the mold cavity called die is the static part.

**Figure 16:**
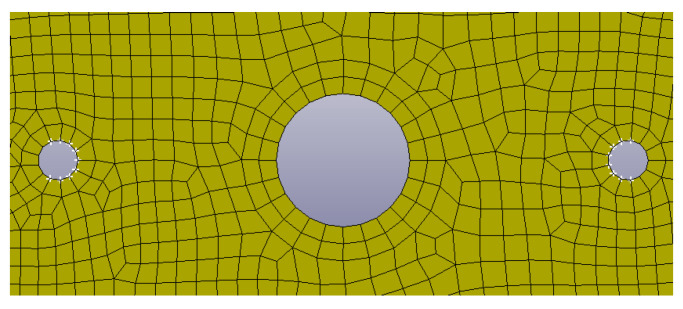
Particular of the model with the constrains on the internal edge of the support pin holes.

The contact model (* CONTACT_FORMING_ONE_WAY_SURFACE_TO_SURFACE) between the mold and the laminate was set with the experimental values of the frictional coefficient obtained by the tool-ply test. The laminate was reproduced as a *PART_COMPOSITE composed by 4 layers (0.25-mm thickness), as the real laminate. For this study, the adopted approach is macroscopic and based on correlation with experimental tests. Furthermore, the two pins of the mold that keep in position the laminate were simulated with constrains on x and y translation on the internal edge of the holes, as reported in Figure 16. The holes were reproduced only for thermoplastic composites because there is not any support in the press mold of thermosetting Double Dome.

### 3.4. Double Dome Bending Test Simulation

**Figure 17:**
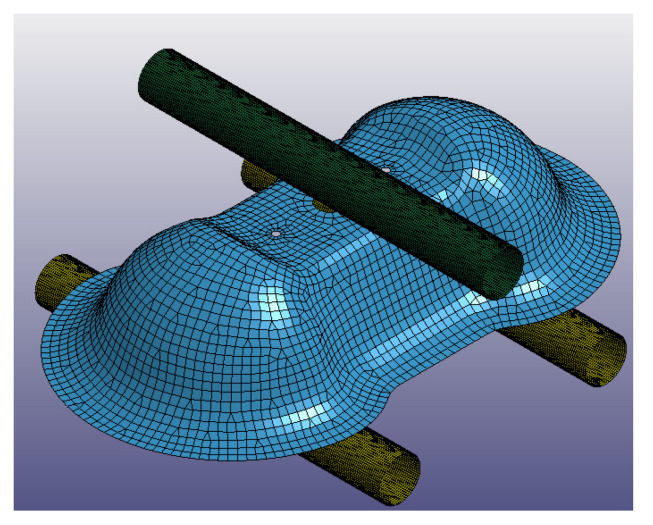
FEA model of three-point bending test on Double Dome.

The three-point bending test on Double Dome has been simulated with a FEA model on LS-DYNA. The steel bars were meshed with element size of 1 mm and modeled with *MAT_RIGID card. The Double Dome was meshed with shell element size of 4 mm, and the *PART_COMPOSITE composed by 4 layers (0.25 mm thickness) was associated with the laminate. The card material assigned to the Double Dome is *MAT_LAMINATED_COMPOSITE_FABRIC (MAT_058), compiled with the parameters obtained by the material card setting on the static tests of the laminate. Figure 17 reports the FEA model of three-point bending test on Double Dome.

### 3.5. Mapping Procedure

**Figure 18:**
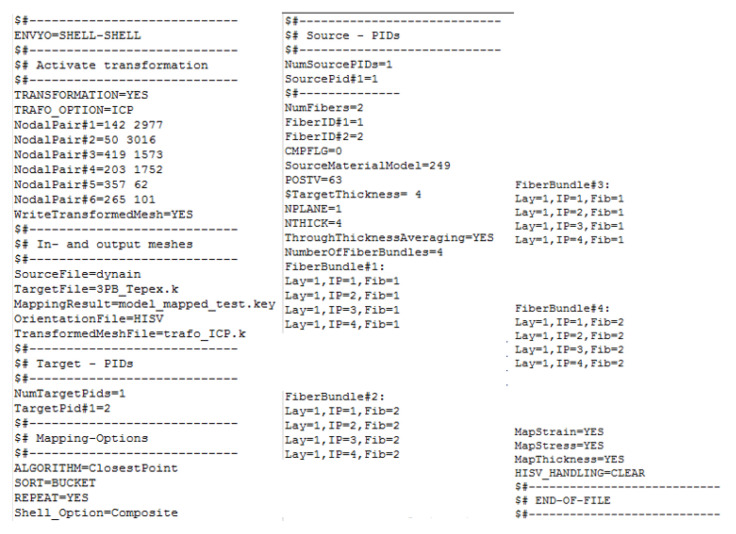
ENVYO—Mapping code.

The Mapping Procedure allows to transfer the results of the thermoforming simulation on another mesh, in this case the structural model for the three-point bending test of the Double Dome. In general, the mesh for the forming simulation and the mesh for the structural simulation are different, so it a specific software that recognizes the shape of the component and transfers element by element the results of the thermoforming simulation is needed, in terms of residual deformations and stresses, shell thickness, and shear angle. The shear angle is an angular value that describes specifically the rotation of fibers with respect to the initial position inside the composite laminate. Together with the other transferred parameters, it allows for taking into account the impact of laminate variation on the mechanical performance. The software used for the Mapping process is Envyo, a software dedicated to map the result data into LS-DYNA meshes, that is developed by DYNAmore (Stuttgart, Germany). Envyo does not have a graphical user interface; it is controlled using a text-based input file (Figure 18). To perform a geometry matching prior to the Mapping process, it is required to select some nodes on the forming model and the structural model to allow Envyo to correctly align the shape on source and target side. For the Double Dome model, it was enough to select six nodes.

## 4. Results and Discussion

### 4.1. Static Tests

**Figure 19:**
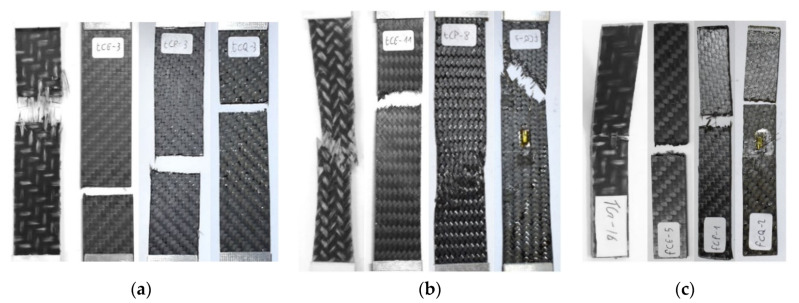
Comparison of different breaking behavior of the four materials (from left to right for each figure: GF/PP, CF/PA66, CF/EP Angeloni, CF/EP Solvay) for the Tensile 0° test (**a**), Tensile 45° test (**b**), and Bending 0° test (**c**).

Figure 19 shows the picture of a significative specimen after the static test, for the studied materials. It can be seen that the GF/PP has a ductile behavior on Tensile 45° and Bending 0° tests because of the PP matrix and the GF weaker rigidity. The other CF laminates showed the same fragile breaking at 0°, while, at 45°, a small transversal necking at break, due to the PA66 and Epoxy matrix high rigidity.

**Figure 20:**
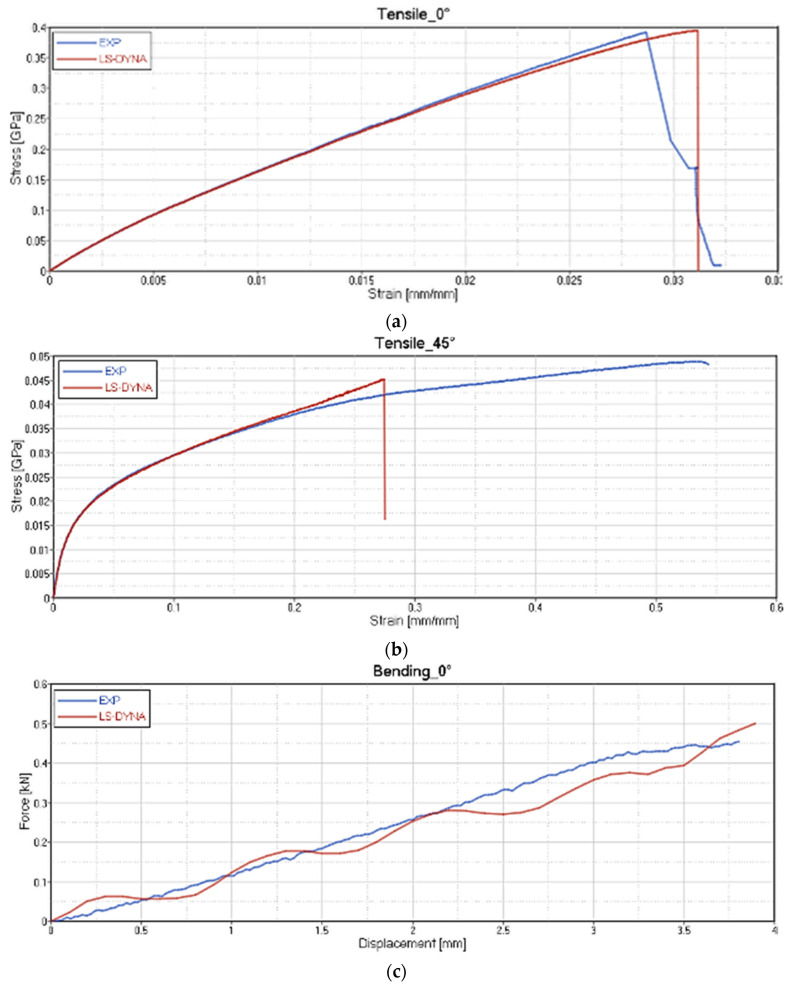
Correlation between experimental data and virtual material card setting for GF/PP. Tensile test at 0° **(a**); Tensile test at 45° (**b**) and Force-Displacement (kN-mm) for Bending test at 0° (**c**).

**Figure 21:**
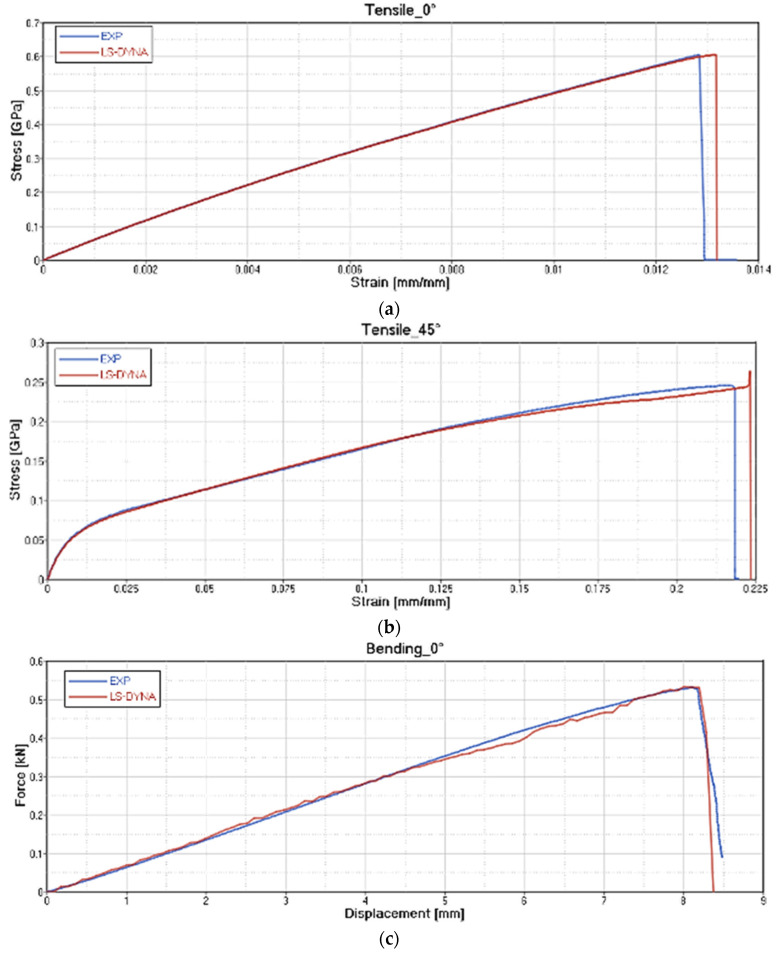
Correlation between experimental data and virtual material card setting for CF/PA66. Tensile test at 0° **(a**); Tensile test at 45° (**b**) and Force-Displacement (kN-mm) for Bending test at 0° (**c**).

**Figure 22:**
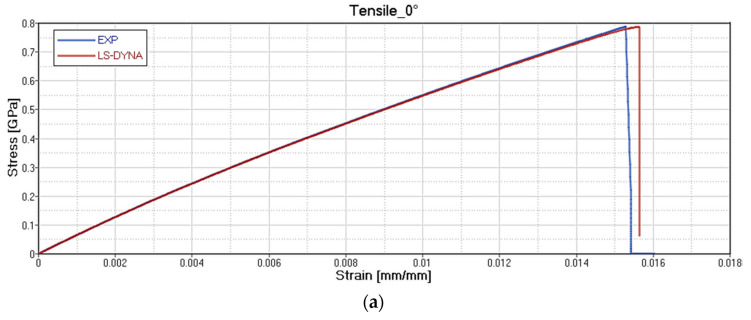

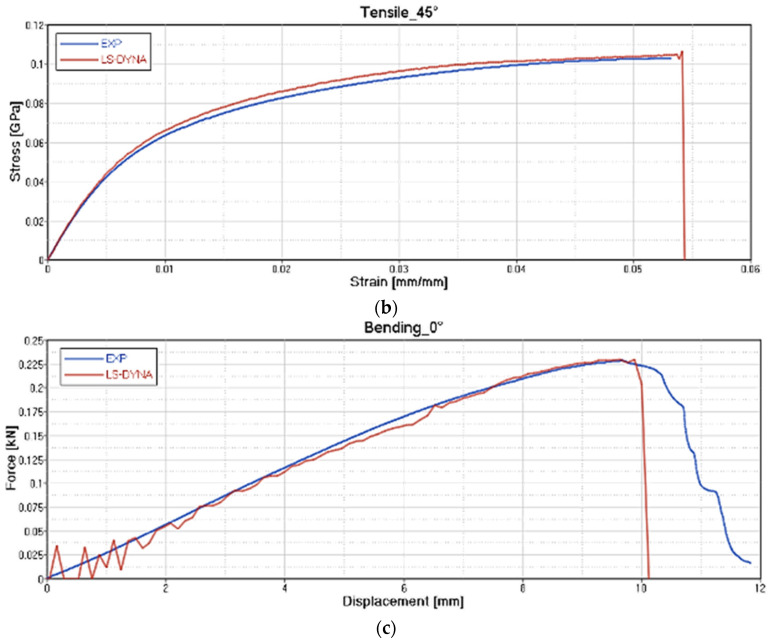
Correlation between experimental data and virtual material card setting for CF/EP-Angeloni. Tensile test at 0° **(a**); Tensile test at 45° (**b**) and Force-Displacement (kN-mm) for Bending test at 0° (**c**).

**Figure 23:**
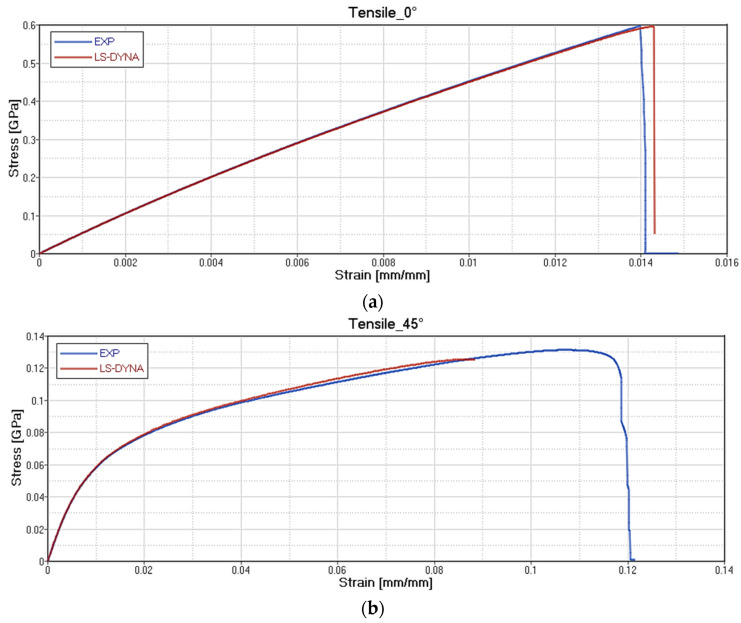

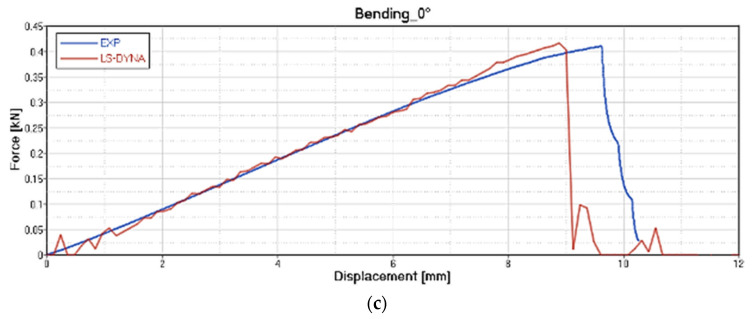
Correlation between experimental data and virtual material card setting for CF/EP-Solvay. Tensile test at 0° **(a**); Tensile test at 45° (**b**) and Force-Displacement (kN-mm) for Bending test at 0° (**c**).

The results of the card setting are reported, respectively, for each type of test on GF/PP (Figure 20), CF/PA66 (Figure 21), CF/EP-Angeloni (Figure 22), and CF/EP-Solvay (Figure 23). Blue lines relate to the experimental data, and red lines to the material card setting. The graphs are intended as Stress-Strain (GPa mm/mm) for Tensile test at 0° (a) and Tensile test at 45° (b), and Force-Displacement (kN-mm) for Bending test at 0° (c). The fitting of tensile 0° and tensile 45° test for CF/PA66 and CF/EP-Angeloni simulates accurately both the Young Modulus and the tensile strength values. The tensile strength at 45° for GF/PP and CF/EP-Solvay was decreased in order to avoid instability phenomena due to too high values of maximum deformation of the elements set on the card material and to obtain a good numerical-experimental correlation of bending 0° test. The reported oscillations are due to explicit modelization, which is subjected to fluctuations for the representation of a dynamic phenomenon.

### 4.2. Process Tests

**Figure 24:**
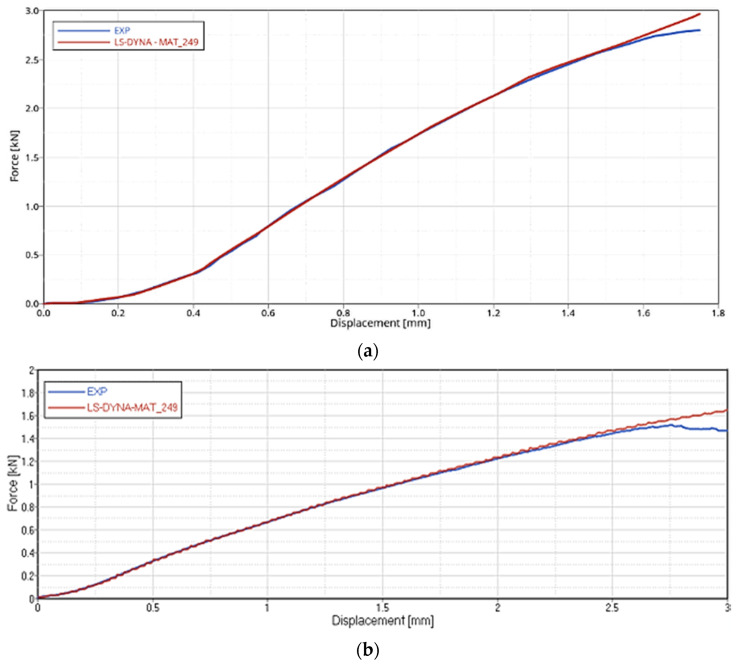
FEA-experimental correlation of the Tensile test on GF (**a**), CF (**b**) dry fabrics.

**Figure 25:**
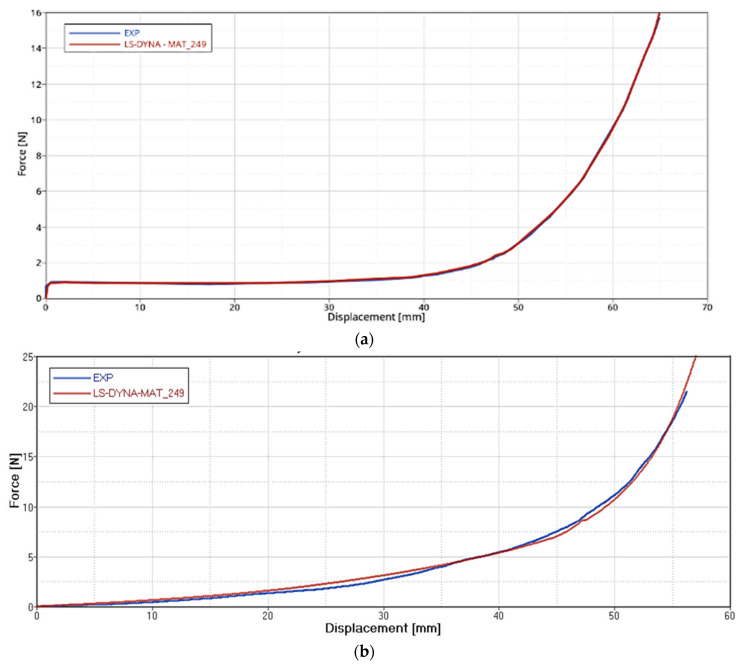
FEA-experimental correlation of the Shear test on GF (**a**), CF (**b**) dry fabrics.

The correlation results for the Intra-ply Tests (Tensile and Shear) on GF and CF fabric, in terms of Force-Displacement, are reported in Figure 24 and Figure 25. Blue lines relate to the experimental data, and red lines to the material card setting. The simulation has a consistent match with the experimental curves.

**Figure 26:**
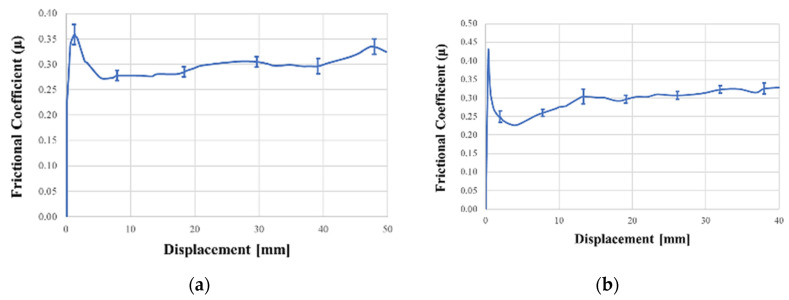
Average results of the tool-ply test: GF/PP (**a**) and CF/PA66 (**b**).

In Figure 26, the average result of the Tool-ply test on three specimens for GF/PP and CF/PA66 is reported in terms of coefficient of Friction-Displacement. The static frictional coefficient calculated at the peak of the curve is about 30% higher than the dynamic frictional coefficient calculated in the plateau. The frictional coefficients obtained in Table 3 were used in the thermoforming simulation to reproduce the tool-ply friction between the external composite ply and the steel of the mold.

### 4.3. Thermoforming Process

**Figure 27:**
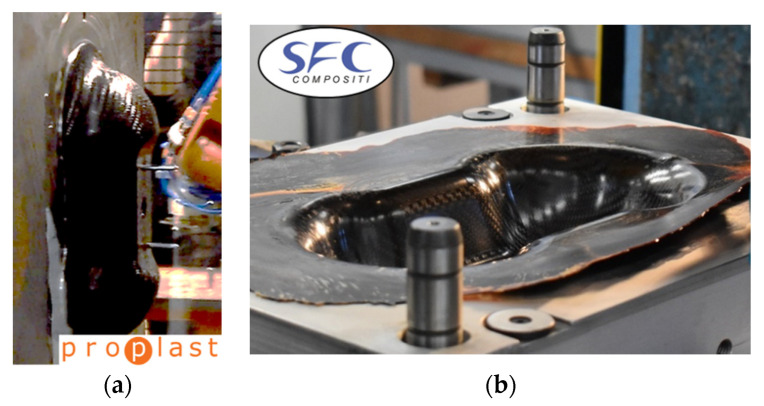
GF/PP and CF/PA66 Double Dome obtained by Horizontal Press (**a**), CF/EP-Angeloni and CF/EP-Solvay Double Dome obtained by Vertical Press (**b**).

**Figure 28:**
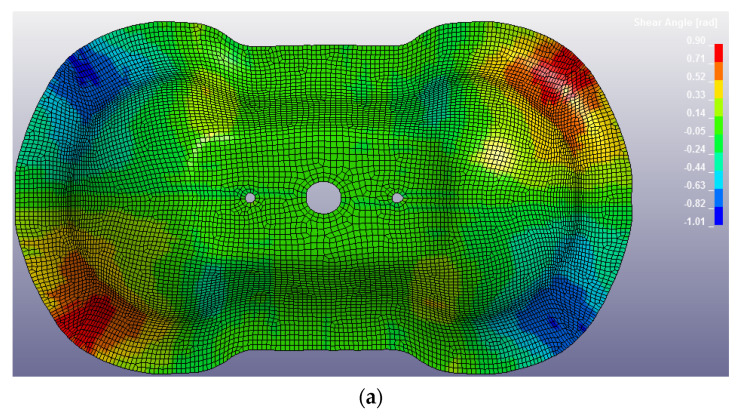

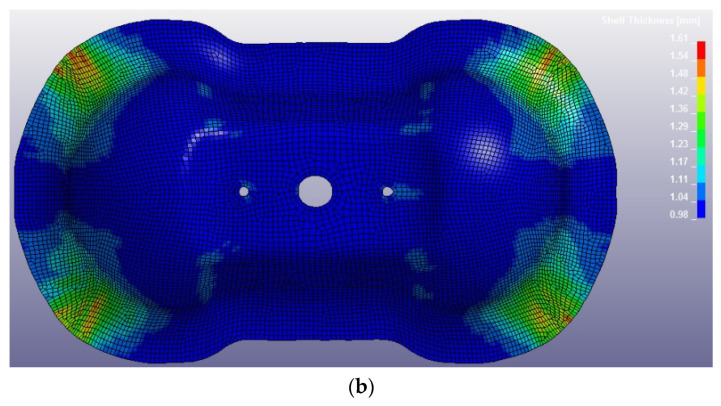
GF/PP Double Dome after thermoforming simulation: values of shear angle (**a**) and values of shell thickness (**b**).

**Figure 29:**
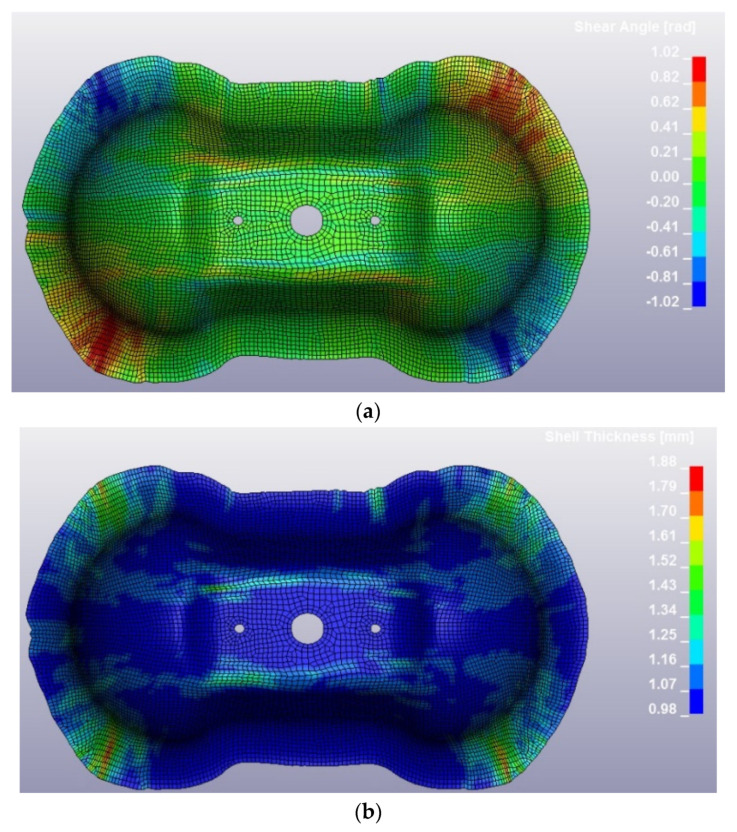
CF/PA66 Double Dome after thermoforming simulation: values of shear angle (**a**) and values of shell thickness (**b**).

The Double Dome was produced by Horizontal Press for thermoplastic organosheets and by Vertical Press for thermosetting prepregs, and the picture of the extraction is reported in Figure 27. The post-process of the thermoforming simulation was focused on the correlation of shear angle and shell thickness on the Double Dome model. The thickness of the laminate is set with a *PART_COMPOSITE composed by 4 layers of ply thickness 0.25 mm. The thickness after thermoforming simulation was obtained by history variables of * MAT_0249. The results are reported respectively in Figure 28 for GF/PP and in Figure 29 for CF/PA66. A general increase of the average shell thickness (about 1.1 mm instead the nominal thickness of 1.0 mm) was observed, but the most deformed areas are the bases of the domes, with higher values of shear angle and shell thickness than other areas. Probably the shear deformation caused by the thermoforming process induces a decrease of the area and a consequent increase of thickness to conserve the volume. This phenomenon was observed on both thermoplastic composites. After parameters optimization, the computational time of thermoforming simulation was reduced to three hours.

**Figure 30:**
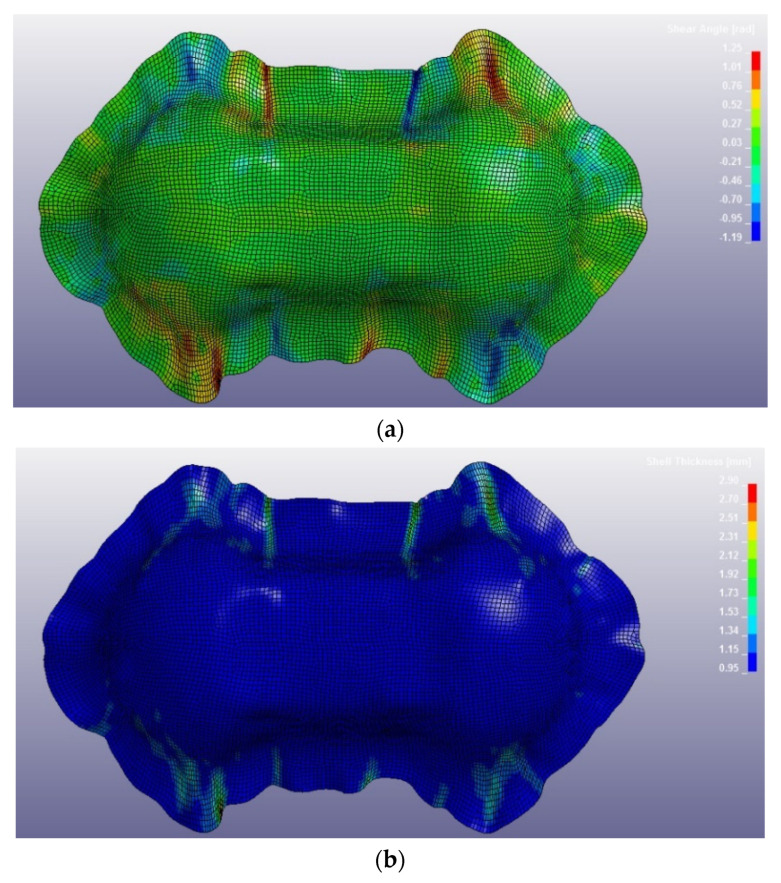
CF/EP-Angeloni and CF/EP-Solvay Double Dome after thermoforming simulation: values of shear angle (**a**) and values of shell thickness (**b**).

Figure 30 reports the thermoforming simulation results, which are the same for CF/EP-Angeloni and CF/EP-Solvay. In this case, a slight decrease of shell thickness was observed (0.95 mm instead 1 mm). It is possible that the molding phase of the production process caused a partial leakage of resin from the mold, which accumulated at the laminate edges. This phenomenon was also observed in the real process, and the deformed edges were trimmed to obtain the final shape. In fact, the thickness experimentally measured for the thermosetting Double Dome revealed about 0.8 mm.

### 4.4. Structural Bending Test

**Figure 31:**
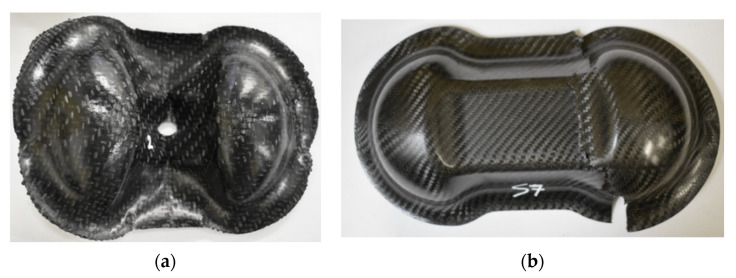
Double Dome specimens in GF/PP (**a**) and CF/EP-Solvay (**b**) after the bending test.

The structural simulation of the three-point bending test was performed both with and without the results of the thermoforming simulation, in order to study the effect of the Mapping on the structural simulation. As can be seen in Figure 31, the GF/PP Double Dome showed a ductility already seen in the static characterization, keeping the final deformation at the end of the test, folding but not really breaking. For the CF Double Dome, instead, the fiber breaking has been noted in the central planar zone, carrying to the final split of the Domes for the CF/EP-Solvay.

**Figure 32:**
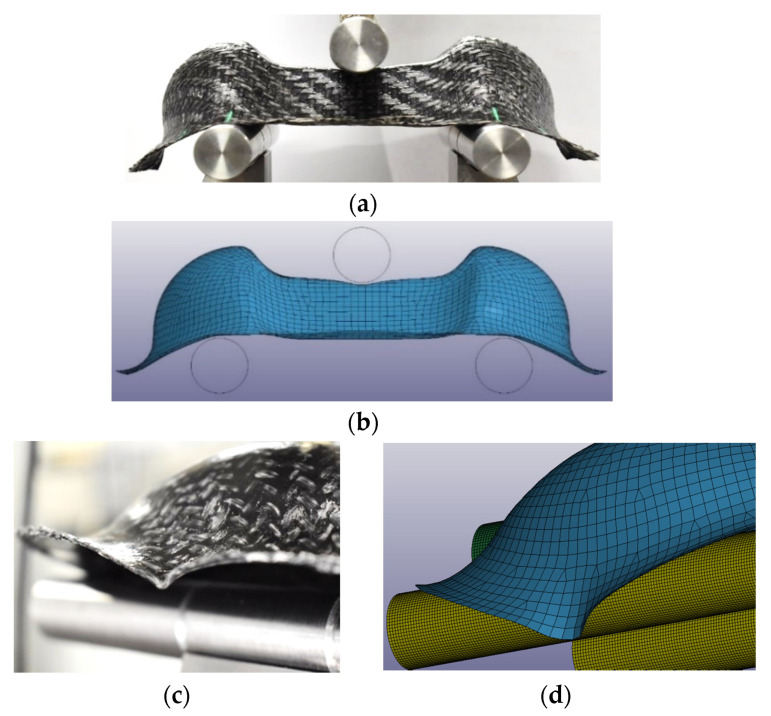
Experimental–FEA visual comparison during the three-point bending test on the GF/PP Double Dome: global view (**a**,**b**) and a detail on the bases of the dome (**c**,**d**).

**Figure 33:**
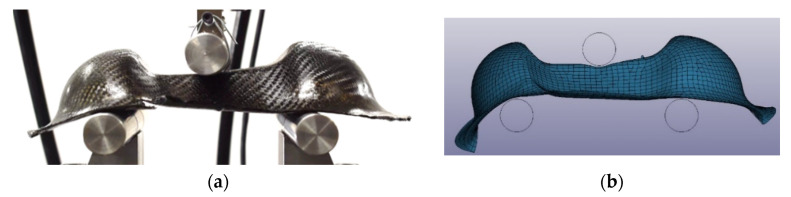
Experimental–FEA visual comparison during the three-point bending test on the CF/PA66 Double Dome: experimental test (**a**) and virtual simulation (**b**).

Figure 32 and Figure 33 show the visual comparisons between the experimental test and the simulation with Mapping for GF/PP and CF/PA66. The simulation reproduces quite well the local deformations to which the Double Dome is subjected during the test.

**Figure 34:**
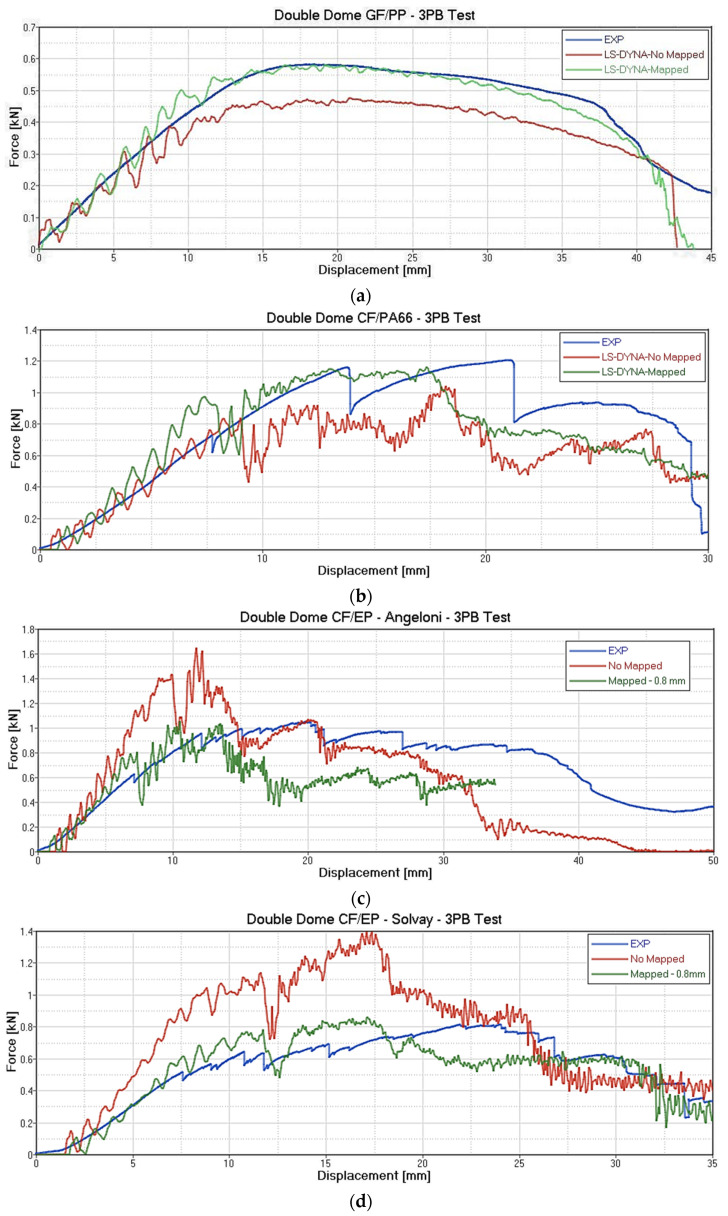
FEA-experimental correlation of the three-point bending test on GF/PP (**a**), CF/PA66 (**b**), CF/EP-Angeloni (**c**), and CF/EP-Solvay Double Dome (**d**).

Figure 34 reports the correlation between the experimental test and the virtual model, for GF/PP, CF/PA66, CF/EP-Angeloni, and CF/EP-Solvay. In blue is the experimental average curve, in red is the FEA no mapped curve, and in green is the FEA mapped curve. The bending maximum resistance is different for the composites studied. The GF/PP is the lowest because of the GF weaker than CF. The CF/PA66 is higher than CF/EP because of the maintaining of the thickness during the process. The CF/EP-Angeloni is higher than CF/EP-Solvay, depending on the major fragility of Solvay resin, demonstrated already in the static tests.

The simulation without Mapping underrates the maximum load of about 15% for GF/PP and 20% for CF/PA66. Instead, the simulation with Mapping follows the trend of the experimental curve up to the maximum. The progressive breaks of the experimental curve of CF/PA66 are due to local defects that cannot be reproduced by the simulation. The simulation without Mapping on CF/EP-Angeloni and CF/EP-Solvay overestimates the maximum load about 60% for both materials because it does not take into account the leakage of resin from the mold and the consequent reduction in thickness of the laminate. Instead, the simulation with Mapping follows the trend of the experimental curves up to the maximum, with an appropriate correction of the average thickness (0.8 mm) based on the experimental measurement carried out after the thermoforming correlation.

## 5. Conclusions

The outstanding advantages of the press molding composite materials in the actual manufacturing industry is relevant in terms of reducing the production cycle time and the energy saving, enhancing the surface quality requirements and the final mechanical performances of the molded part, in comparison to the traditional vacuum-bag autoclave process. New composite laminates, like organosheets and fast-curing prepregs, have to be accurately validated in order to replace the traditional prepregs. Furthermore, the forecasting of the final performance of the manufactured part is an interesting objective to achieve.

The forming process of the Double Dome case study was evaluated in this study on thermoplastics and thermosets composites laminates, using horizontal and vertical press, to give an industrial validity to the project. The materials were experimentally characterized to properly set a material card on a FEA model. The forming process was reproduced using LS-DYNA, including the models of molding parts and composite laminate. The variables resulting from the process simulation were transferred to a new model using the ENVYO Mapping procedure. The new model was used to correlate a structural three-point bending test on Double Dome, in order to take into account the influence of the manufacturing process on the final performance of the part.

This paper highlighted that the ENVYO Mapping process improves the reliability of the correlation on the structural test of the finished part. In particular, for thermoplastics organosheets, it was verified experimentally and reproduced virtually an increase of shell thickness and a consequent increase of maximum load. Instead, for thermosetting composite laminates, the forming simulation underrates the resin leaking from the mold. For this reason, it is necessary to set the final thickness in order to simulate the real thickness of the produced part in thermoset resin. Possible next steps are to improve the simulation of thickness variation (in particular for thermosets composites) and ply-ply interface behaviors in order to verify this approach on other geometries and automotive components.

## Figures and Tables

**Table 1:** Thermoforming parameters for the chosen materials.

Material	Molding Process	Temperature (°C)	Pressure (bar)	Cycle Time (s)
GF/PP	Horizontal Press	180	1213	52
CF/PA66	Horizontal Press	290	1503	50
CF/EP—Angeloni	Vertical Press	140	90	480
CF/EP—Solvay	Vertical Press	140	70	300

**Table 2:** Static characterization: Specifications of the experimental tests.

Type of Test	Layers Orientation	Number of Specimens	Test Speed (mm/min)	Specimen Dimensions (mm)	Standard Reference
Tension	[0] _8_	5	2	200 × 25 × 2	ASTM D3039
Tension	[45] _8_	5	2	200 × 25 × 2	ASTM D3518
Four-point Bending	[0] _8_	5	2	100 × 15 × 2	ASTM D7264

**Table 3:** Static and Dynamic Frictional coefficients calculated for GF/PP and CF/PA66.

Material	Temperature (°C)	Static Frictional Coefficient μ_s_ (/)	Dynamic Frictional Coefficient μ_d_ (/)
GF/PP	180	0.35	0.30
CF/PA66	290	0.42	0.30
